# Early Combination of Albumin With Crystalloid Administration Might Reduce Mortality in Patients With Cardiogenic Shock: An Over 10-Year Intensive Care Survey

**DOI:** 10.3389/fcvm.2022.879812

**Published:** 2022-05-27

**Authors:** Zhi-ye Zou, Bin Wang, Wen-jun Peng, Zhi-peng Zhou, Jia-jia Huang, Zhen-jia Yang, Jing-jing Zhang, Ying-yi Luan, Biao Cheng, Ming Wu

**Affiliations:** ^1^Department of Critical Care Medicine and Hospital Infection Prevention and Control, Shenzhen Second People's Hospital & First Affiliated Hospital of Shenzhen University, Shenzhen, China; ^2^Department of Ultrasound, Longgang Central Hospital of Shenzhen, Shenzhen, China; ^3^Department of Cardiovascular, Longgang Central Hospital of Shenzhen, Shenzhen, China; ^4^Postgraduate Education, Shantou University Medical College, Shantou, China; ^5^Department of Central Laboratory, Beijing Obstetrics and Gynecology Hospital, Capital Medical University, Beijing, China; ^6^Department of Plastic Surgery, General Hospital of Southern Theatre Command of People's Liberation Army, Guangzhou, China; ^7^Graduate School, GuangXi University of Chinese Medicine, Nanning, China; ^8^Department of Critical Care Medicine, The First Dongguan Affiliated Hospital, Guangdong Medical University, Dongguan, China

**Keywords:** cardiogenic shock, fluid therapy, albumins, mortality, early combination

## Abstract

**Background:**

In updated international guidelines, combined albumin resuscitation is recommended for septic shock patients who receive large volumes of crystalloids, but minimal data exist on albumin use and the optimal timing in those with cardiogenic shock (CS). The objective of this study was to evaluate the relationship between resuscitation with a combination of albumin within 24 h and 30-day mortality in CS patients.

**Methods:**

We screened patients with CS from the Medical Information Mart for Intensive Care IV (MIMIC-IV) database. Multivariable Cox proportional hazards models and propensity score matching (PSM) were employed to explore associations between combined albumin resuscitation within 24 h and 30-day mortality in CS. Models adjusted for CS considered potential confounders. *E*-value analysis suggested for unmeasured confounding.

**Results:**

We categorized 1,332 and 254 patients into crystalloid-only and early albumin combination groups, respectively. Patients who received the albumin combination had decreased 30-day and 60-day mortality (21.7 vs. 32.4% and 25.2 vs. 34.2%, respectively, *P* < 0.001), and the results were robust after PSM (21.3 vs. 44.7% and 24.9 vs. 47.0%, respectively, *P* < 0.001) and following *E*-value. Stratified analysis showed that only ≥ 60 years old patients benefited from administration early albumin. In the early albumin combination group, the hazard ratios (HRs) of different adjusted covariates remained significant (HRs of 0.45–0.64, *P* < 0.05). Subgroup analysis showed that resuscitation with combination albumin was significantly associated with reduced 30-day mortality in patients with maximum sequential organ failure assessment score≥10, with acute myocardial infarction, without an Impella or intra-aortic balloon pump, and with or without furosemide and mechanical ventilation (HRs of 0.49, 0.58, 0.65, 0.40, 0.65 and 0.48, respectively; *P* < 0.001).

**Conclusion:**

This study found, compared with those given crystalloid-only, resuscitation with combination albumin within 24 h is associated with lower 30-day mortality of CS patients aged≥60. The results should be conducted to further assess in randomized controlled trials.

## Introduction

Cardiogenic shock (CS) is a clinical syndrome characterized by significantly reduced cardiac output, whereby an extreme decline in cardiac function may result in severe acute peripheral circulatory failure (1). Despite significant advances in CS therapy in recent years, namely, with the gradual application of the intra-aortic balloon pump (IABP), percutaneous coronary intervention (PCI), the Impella pump and extracorporeal membrane oxygenation (ECMO), the clinical mortality of CS still exceeds 40% (2).

Prompt and effective treatment is crucial for patients with CS. Aetiological treatment, timely use of vasoactive drugs, and mechanical assistance have been recognized and confirmed by clinical studies (3, 4). In addition, intravenous infusion for patients with CS is primarily based on pathophysiological considerations. According to current guidelines, fluid resuscitation should be considered as first-line treatment unless there are clear signs of fluid overload (4). However, the specific fluid type of resuscitation is not specified in the latest guidelines. Furthermore, there is limited literature on the type of fluid required for resuscitation in patients with CS.

Many previous studies have found no association with lower mortality in the intensive care unit (ICU) among patients with severe sepsis or septic shock receiving colloid therapy compared with crystalloids (5, 6). Nevertheless, a recent retrospective study using propensity score matching (PSM) to adjust confounding and restricted mean survival time (RMST) analysis showed that early combined use of albumin might improve the survival rate of patients with sepsis (7), and the heterogeneity of septic shock might be responsible for disparate findings. The updated international guidelines suggest using albumin in adult patients who receive large volumes of crystalloids for septic shock (8). Albumin is the most abundant plasma protein and has many biological functions, including binding to endo- and exogenous substances (9), antithrombotic effects (10) and maintenance of colloidal osmotic pressure (11). Based on different clinical types of shock, the pathophysiological characteristics of patients also differ, and it remains unclear whether combined albumin resuscitation can improve the prognosis of CS patients. Therefore, to evaluate the effectiveness of albumin for patients with CS, we used the Medical Information Mart for Intensive Care IV (MIMIC-IV) database to assess whether resuscitation with combination albumin within 24 h after CS onset was associated with lower 30-day mortality.

## Materials and Methods

### Study Design, Data Source and Participants

We performed a retrospective cohort study using MIMIC-IV version 1.0 (12) data (https://physionet.org/content/mimiciv/1.0/), which are sourced from two inpatient database systems: the customized hospital Electronic Medical Record System (EHR) and the clinical information system of the intensive care unit (ICU). MIMIC-IV contains 256,878 patients from 2008 to 2019 and includes demographic, vital sign, laboratory test, and drug data, among others. The database is an upgraded version of MIMIC-III with high-quality critical medical data. Author ZOU (Certification number 35951237) has completed the Collaborative Institutional Training Initiative examination and can access the MIMIC-IV database.

The inclusion criteria were in accordance with ICD-9 (785.51 and 998.01) and ICD-10 (R57.0 and T81.11XA) diagnosis codes and patients who were resuscitated within 24 h after CS onset. The exclusion criteria were as follows: age <18 years old; data from multiple intensive care unit (ICU) admission other than the first admission; secondary diagnosis of liver disease, solid tumor, or other cancer on admission(ICD-9 or ICD-10); ICU stay <24 h; albumin preceded crystalloid; combined albumin >24 h; no crystalloid given. For patients admitted to the ICU multiple times, we only included data from the first admission.

### Research Procedures and Definitions

We categorized patients into a crystalloid-only group and an early combination group (crystalloid combined with albumin in the first 24 h); we extracted data using a structure query language (SQL) with Navicat Premium tools (version 12.1.12). The following data were recorded: age, sex, ethnicity, insurance, weight, probable etiologies, history of diseases, Charlson comorbidity index (CCI), vital signs on the first day, the first day of maximum Sequential Organ Failure Assessment (SOFA) score and Simplified Acute Physiology Score II (SAPS) score, laboratory findings, central venous pressure (CVP), in-hospital management and medication, stage of acute kidney injury (AKI) (13) and fluid type during ICU stay. In-hospital management included percutaneous coronary intervention (PCI), intra-aortic balloon pump (IAPB), Impella pump, mechanical ventilation, and renal replacement therapy (RRT). Probable etiologies included acute myocardial infarction (AMI) (14) and acute heart failure (AHF) (15). Dopamine, norepinephrine, and their total usage time were collected as in-hospital medication data. In this study, crystalloids included both normal saline and lactated Ringer's (LR) solution; albumin included 5% and 25% human serum albumin (HSA). The main outcome was mortality including 30-day mortality and 60-day mortality.

### Statistical Analysis

Data are presented as the mean ± standard deviation (mean ± SD), median and interquartile interval (median, IQR), or percentage (*n*%), as appropriate. Comparisons between the crystalloid-only group and early combination group were performed using Student's t test, the Mann–Whitney test, and the χ^2^ test, as appropriate. Risk factors affecting clinical outcomes, including etiology (AMI), in-hospital management (IABP or IMPELLA) and medication (furosemide), and disease severity (SOFA), were examined for an association with 30-day mortality using a Cox proportional hazards model. An extended Cox model was applied to adjust for covariates that might affect outcomes. We also explored the potential for unmeasured confounding between early resuscitation with albumin and 30-day mortality by calculating *E*-values (16). The *E*-value quantifies the required magnitude of an unmeasured confounder that might negate the observed association between combined albumin and 30-day mortality.

Propensity score matching (PSM) was selected to balance confounding factors. Variables including age, sex, ethnic group, insurance condition, weight, probable etiology, history of the disease, CCI, vital signs on the first day, the first day of maximum SOFA and SAPS scores, and in-hospital management and medication were chosen to generate the PS based on clinical significance and previous literature. We used a multivariable logistic regression model. A one-to-one nearest neighbor matching algorithm was applied using a caliper width of 0.2. After PSM, we used the standardized mean difference (SMD) and *P*-value to judge the balance of essential characteristics between the groups. When the SMD of a variable is larger than 0.1, it can be considered an imbalance between groups (17). Finally, 253 patients in each group were well matched, and their data were extracted for further analyses. We also performed analysis of interaction between age and early albumin administration with a Cox proportional hazards model and performed PSM analysis for CS patients with no albumin deficiency on day1. Risk factors for 30-day mortality were also evaluated, and the analysis was conducted in the population after PSM. Survival analysis for patients with and without albumin treatment was performed using Kaplan–Meier (K–M) analysis and log-rank tests before and after PSM.

Subgroup and interaction analyses were carried out to determine whether albumin administration and 30-day mortality differ across various subgroups classified by SOFA, AMI, IABP, furosemide, and mechanical ventilation. Subgroup analysis also used a Cox model adjusted for all variables included in patient baseline information. Average values were used to replace the missing values of some variables. Multiple imputations were used for missing values under the assumption of missing at random (18). Two-tailed *P* values < 0.05 were considered to indicate statistical significance. All statistical analyses were performed using Stata 15.1 (StataCorp, College Station, TX, USA), Empower (R; www.empowerstats.com; X&Y Solutions Inc.) and R 4.0.1 software for Windows.

## Results

### Baseline Characteristics

**Figure 1:**
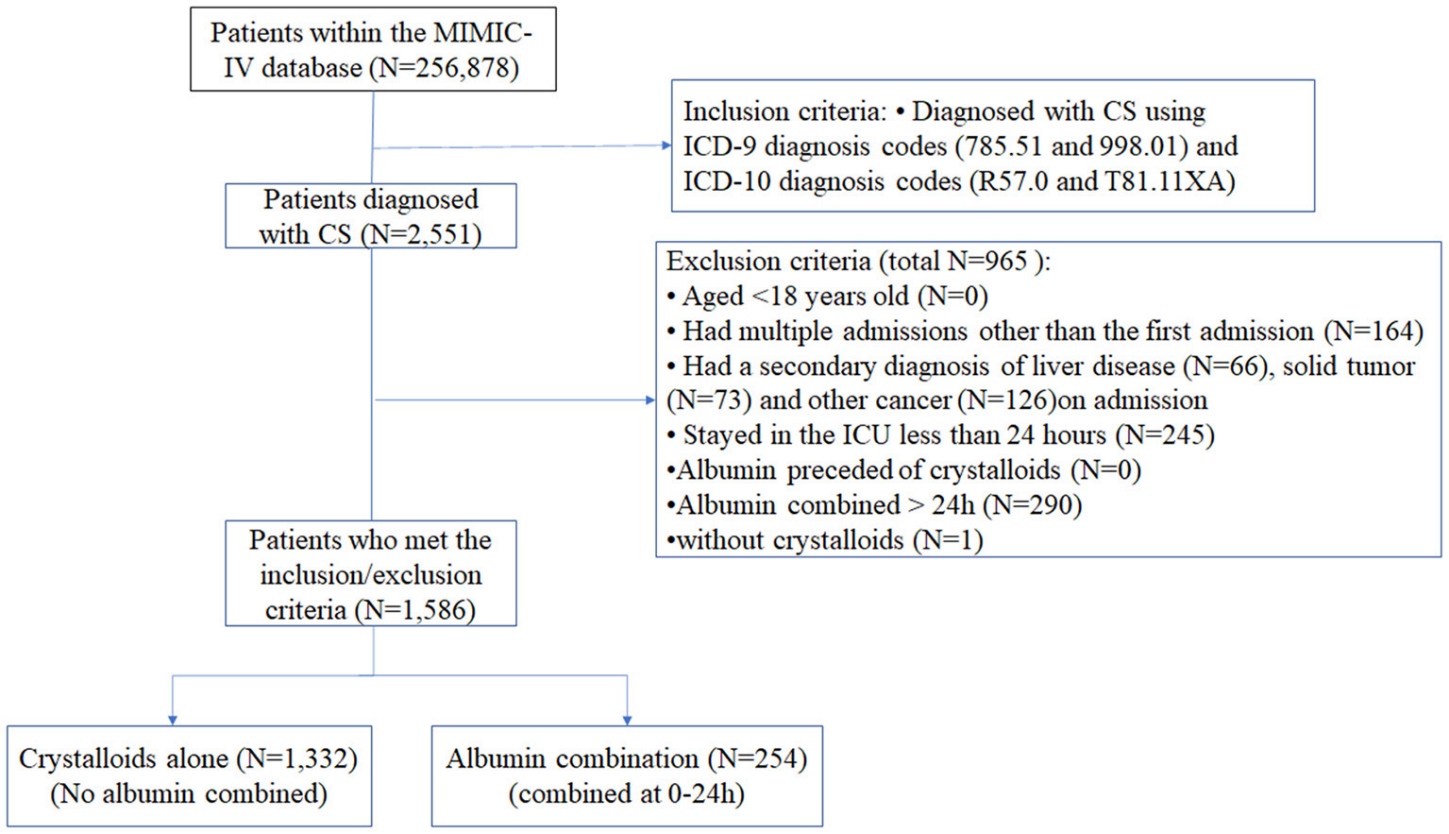
Flow chart of the study.

Data for 2,551 patients with CS were evaluated, and 1,586 patients were included in the study (Figure 1). For fluid resuscitation in the first 24 h, 1,332 patients were given crystalloid only, whereas 254 patients were treated with crystalloid combined with albumin. The missing values of variables were shown in Supplementary Table 1.

In total, 1,586 patients with CS were included in this study (Table 1). The median (IQR) age was 72.2 (61.9, 81.1) years, 60.2% were male, 64.5% were white, and 49.2% had AMI. The crystalloid-only group had significantly higher AMI (51.7 vs. 36.2% *P* < 0.001), AHF, PCI, furosemide, dopamine, urine output, minimum SBP, and albumin (3.3 vs. 2.7g/dl, *P* < 0.001) on the first day than that in the early combination group. Compared to the crystalloid-only group, patients with higher SAPS II and SOFA scores were likelier to be given combined albumin in the first 24 h.

**Table 1:** Baseline characteristics between the two groups before and after propensity score matching (PSM).

	**Propensity score matching**							
		**Before**			**After**			
**Variables**	**Total population** **(*n* = 1,586)**	**Crystalloids only** **(*n* = 1,332)**	**Early combination** **(*n* = 254)**	**P value**	**Crystalloids only (*n* = 253)**	**Early combination** **(*n* = 253)**	**P value**	**SMD**
Age (year), median (IQR)	72.2 (61.9, 81.1)	72.3 (61.9, 81.9)	71.4 (61.8, 78.2)	0.063	71.07 (59.47, 81.82)	71.51 (62.01, 78.18)	0.65	0.030
Male, *n* (%)	954 (60.2)	793 (59.5)	161 (63.4)	0.25	159 (62.8)	160 (63.2)	0.93	0.008
White, *n* (%)	1,023 (64.5)	851 (63.9)	172 (67.7)	0.24	166 (65.6)	171 (67.6)	0.64	0.042
Insurance, medicare, *n* (%)	851 (53.7)	715 (53.7)	136 (53.5)	0.97	131 (51.8)	136 (53.8)	0.66	0.040
Weight (kg), median (IQR)	79.9 (67.0, 93.7)	79.9 (66.7, 94.0)	80.0 (69.5, 91.1)	0.40	77.8 (63.4, 93.0)	80.0 (69.5, 91.0)	0.13	0.086
Etiology, *n* (%)								
AMI	781 (49.2)	689 (51.7)	92 (36.2)	<0.001	92 (36.4)	92 (36.4)	1.00	<0.001
AHF	997 (62.9)	899 (67.5)	98 (38.6)	<0.001	94 (37.2)	98 (38.7)	0.71	0.033
History of disease, *n* (%)								
Hypertension	1,232 (77.7)	1,016 (76.3)	216 (85.0)	0.002	208 (82.2)	215 (85.0)	0.40	0.075
Diabetes	642 (40.5)	544 (40.8)	98 (38.6)	0.50	92 (36.4)	98 (38.7)	0.58	0.049
Chronic pulmonary disease	510 (32.2)	432 (32.4)	78 (30.7)	0.59	85 (33.6)	78 (30.8)	0.51	0.059
CKD	655 (41.3)	566 (42.5)	89 (35.0)	0.027	95 (37.5)	89 (35.2)	0.58	0.049
Stroke	193 (12.2)	152 (11.4)	41 (16.1)	0.035	42 (16.6)	40 (15.8)	0.81	0.021
Vital signs at 1st day, median (IQR)								
Minimum SBP (mmHg)	81.0 (72.0, 88.0)	81.0 (72.0, 89.0)	80.0 (70.0, 85.5)	0.004	79.0 (70.0, 86.0)	80.0 (70.0, 85.0)	0.62	0.024
Minimum DBP (mmHg)	43.0 (36.0, 50.0)	43.0 (36.0, 50.0)	42.0 (36.0, 48.0)	0.12	43.0 (34.0, 49.0)	42.0 (36.0, 48.0)	0.81	0.094
Scoring system, mean (SD)								
CCI	6.9 (2.5)	6.9 (2.5)	6.5 (2.4)	0.009	6.40 (2.64)	6.50 (2.39)	0.66	0.039
Maximum SOFA at 1st day	8.6 (4.1)	8.2 (4.1)	10.4 (3.5)	<0.001	10.6 (4.0)	10.4 (3.4)	0.52	0.057
Maximum SAPS II at 1st day	45.2 (14.9)	44.7 (15.3)	47.5 (12.7)	0.006	48.6 (16.5)	47.5 (12.8)	0.41	0.073
Laboratory findings								
Minimum albumin at 1st day (g/dl), mean (SD)	3.1 (0.6)	3.3 (0.6)	2.7 (0.8)	<0.001	2.97 (0.57)	2.71 (0.78)	0.011	NA
Maximum CVP at 1st day (mmHg), median (IQR)	22.0 (20.0, 29.0)	21.0 (20.0, 30.0)	23.0 (20.0, 27.0)	0.016	21.0 (20.0, 28.0)	23.0 (20.0, 27.0)	0.12	NA
In-hospital management, *n* (%)								
PCI	399 (25.2)	353 (26.5)	46 (18.1)	0.005	44 (17.4)	46 (18.2)	0.82	0.021
IABP	93 (5.9)	84 (6.3)	9 (3.5)	0.086	11 (4.3)	9 (3.6)	0.65	0.041
IMPELLA	71 (4.5)	58 (4.4)	13 (5.1)	0.59	12 (4.7)	13 (5.1)	0.84	0.018
In-hospital medication								
Furosemide at 1st day, *n* (%)	802 (50.6)	723 (54.3)	79 (31.1)	<0.001	72 (28.5)	79 (31.2)	0.50	0.060
Dopamine at 1^st^ day, *n* (%)	316 (19.9)	303 (22.7)	13 (5.1)	<0.001	12 (4.7)	13 (5.1)	0.84	0.018
Duration of dopamine (h), median (IQR)	19.0 (4.0, 50.0)	19.0 (5.0, 50.0)	13.0 (3.5, 49.0)	0.66	19.0 (7.0, 34.0)	13.0 (3.5, 49.0)	0.81	NA
Norepinephrine at 1st day, n (%)	841 (53.0)	658 (49.4)	183 (72.0)	<0.001	187 (73.9)	182 (71.9)	0.62	0.044
Duration of norepinephrine (h), median (IQR)	38.5 (16.0, 76.0)	37.0 (14.0, 74.0)	46.0 (22.0, 95.0)	0.004	45.0 (18.0, 77.0)	45.5 (22.0, 93.0)	0.26	NA

*IQR, interquartile range; AMI, acute myocardial infarction; AHF, acute heart failure; CKD, chronic kidney disease; SBP, systolic blood pressure; DBP, diastolic blood pressure; CCI, charlson comorbidity index; SOFA, sequential organ failure assessment; SAPS II, Simplified acute physiology score II; CVP, central venous pressure; PCI, percutaneous coronary intervention; IABP, intra-aortic balloon pump; NA, not applicable*.

### Outcomes

In PSM, 253 pairs of patients were matched by a 1:1 matching algorithm (Table 1 and Supplementary Figure 1). There was no significant difference between the two groups after PSM, and all SMDs were <0.01.

Patients who received the albumin combination had decreased 30-day mortality and 60-day mortality (21.7 vs. 32.4% and 25.2 vs. 34.2%, respectively, *P* < 0.001), and the results were robust after PSM (21.3 vs. 44.7% and 24.9 vs. 47.0%, respectively, *P* < 0.001; Table 2). The length of stay in ICU (10.61 vs. 5.96 days; *P* < 0.001) and hospital (10.61 vs. 5.96 days; *P* < 0.001) were significantly longer in the early combination group than in the crystalloids group. In the combination group, the percentage of received mechanical ventilation (95.7 vs. 57.1% *P* < 0.001) was significantly higher than in the crystalloids group. Compared with Age <50 years in the crystalloid-only group, the results showed that only≥60 years old patients benefited from early albumin administration (Table 3). In extended multivariable Cox models, we found that the hazard ratio (HR) for different adjusted covariates was continuously significant in the early combination group (HR range 0.45–0.64, *P* < 0.05 for six models; Table 4). In Risk factors analysis showed early combination treatment remained a protective factor for 30-day mortality after PSM (HR 0.390, 95% CI 0.282–0.539; Supplementary Table 2). The primary findings were robust unless an unmeasured confounder existed regarding a lower relative risk of 30-day mortality, with an HR higher than 3.22 with *E*-value (Supplementary Text, Sensitivity Analysis Section)

**Table 2:** Association between early albumin combination and outcomes in cardiogenic shock patients before and after PSM.

**Clinical outcomes**	**Crystalloids only**	**Early combination**			**Crystalloids only**	**Early combination**		
	**Pre-matched cohort**				**Post-matched cohort**			
	***n* = 1,332**	***n* = 254**	**ARR (95%CI)**	***P*-Value**	***n* = 253**	***n* = 253**	**ARR (95%CI)**	***P*-Value**
Minimum albumin at 2nd day (g/dl), mean (SD)	3.0 (0.5)	3.0 (0.6)	NA	0.35	2.8 (0.5)	3.0 (0.6)	NA	0.019
Maximum CVP at 2nd day (mmHg), median (IQR)	20.0 (19.0, 26.0)	24.0 (20.0, 29.0)	NA	<0.001	20.0 (18.0, 25.0)	24.0 (20.0, 29.5)	NA	<0.001
Urine output (ml/1st 24 h), median (IQR)	1,553.5 (775.0, 2,830.0)	1,280.0 (704.5, 2,025.0)	NA	0.001	1,107.0 (400.0, 2,320.0)	1,285.0 (710.0, 2,040.0)	NA	0.18
Volume of crystalloid (ml/1st 24 h), median (IQR)	2,050.0 (843.9, 4,011.7)	4,409.2 (2,621.7, 6,682.5)	NA	<0.001	3,626.6 (1,790.0, 5,643.1)	4,458.5 (2,621.7, 6,682.5)	NA	<0.001
ICU LOS (days), mean (SD)	5.92 (5.34)	10.61 (11.34)	NA	<0.001	5.96 (5.31)	10.61 (11.36)	NA	<0.001
Hospital LOS (days), mean (SD)	11.5 (9.4)	17.7 (17.4)	NA	<0.001	10.05 (8.52)	17.72 (17.42)	NA	<0.001
AKI, *n* (%)	910 (68.3)	196 (77.2)	8.85(3.11, 14.58)	0.005	177 (70.0)	195 (77.1)	7.11(0.55, 14.78)	0.070
AKI stage 3, *n* (%)	247 (18.5)	51 (20.1)	1.54(3.82, 6.89)	0.57	54 (21.3)	51 (20.2)	1.19(-5.88, 8.25)	0.74
RRT at 1st day, *n* (%)	85 (6.4)	12 (4.7)	1.66 (−1.26, 4.58)	0.31	26 (10.3)	11 (4.3)	5.93(1.42, 10.44)	0.010
Mechanical ventilation at 1st day, *n* (%)	761 (57.1)	243 (95.7)	−38.54 (−42.19, −34.89)	<0.001	166 (65.6)	228 (90.1)	−24.51 (−31.42, −17.59)	<0.001
30-day mortality, *n* (%)	431 (32.4)	55 (21.7)	10.70 (5.05, 16.36)	<0.001	113 (44.7)	54 (21.3)	23.32(15.38, 31.26)	<0.001
60-day mortality, *n* (%)	455 (34.2)	64 (25.2)	8.96 (3.05, 14.88)	0.005	119 (47.0)	63 (24.9)	22.13 (14.0, 30.27)	<0.001

*ARR, absolute risk reduction; CI, Confidence interval; CVP, central venous pressure; ICU, intensive care unit; LOS, length of stay; AKI, acute kidney injury; RRT, renal replacement therapy; NA, not applicable*.

**Table 3:** Analysis of interaction between age and early albumin administration.

	***N***	**Crude HR (95% CI)**	***P*-Value**	Adjusted HR^*^(95% CI)	***P*-Value**
Crystalloids only					
Age^†^ <50	126	1 (reference)		1 (reference)	
50 ≤ Age <60	155	1.10 (0.65, 1.85)	0.734	1.04 (0.61, 1.79)	0.873
60 ≤ Age <70	303	1.39 (0.88, 2.19)	0.160	0.95 (0.58, 1.56)	0.850
70 ≤ Age <80	356	2.18 (1.41, 3.37)	<0.001	1.24 (0.76, 2.05)	0.391
80 ≤ Age	392	2.37 (1.54, 3.64)	<0.001	1.24 (0.75, 2.04)	0.397
Early combination					
Age <50	16	2.32 (0.95, 5.68)	0.065	1.61 (0.65, 3.96)	0.301
50 ≤ Age <60	39	0.41 (0.13, 1.34)	0.140	0.36 (0.11, 1.17)	0.090
60 ≤ Age <70	57	0.36 (0.12, 1.04)	0.060	0.33 (0.11, 0.97)	0.044
70 ≤ Age <80	91	0.22 (0.08, 0.61)	0.004	0.22 (0.08, 0.62)	0.004
80 ≤ Age	51	0.14 (0.04, 0.43)	0.001	0.12 (0.04, 0.40)	<0.001

* *The analysis of interaction effects used a Cox model adjusted for all variables included in patient baseline information*.

† *Stratification and interaction effects were performed for age*.

**Table 4:** Association between early albumin use and 30-day mortality using an extended model approach.

	**Hazard ratio**	**95% confidence interval**	***P*-Value**
Model 1	0.62	0.46–0.82	0.001
Model 2	0.62	0.47–0.83	0.001
Model 3	0.64	0.48–0.86	0.003
Model 4	0.54	0.39–0.72	<0.001
Model 5	0.47	0.35–0.64	<0.001
Model 6	0.45	0.33–0.62	<0.001

*Adjusted covariates: Model 1 = early albumin use. Model 2 = Model 1 + (History of disease including hypertension, diabetes, chronic pulmonary disease, CKD and stroke). Model 3 = Model 2 + (age gender ethnicity insurance weight). Model 4 = Model 3 + (probable etiology including AMI and AHF). Model 5 = Model 4 + (Scoring system including CCI, SOFA and SAPS II). Model 6 = Model 5 + (In-hospital management and medication including PCI, IABP, IMPELLA and Furosemide at 1st day)*.

As the albumin values for 59.33% (941/1,586) patients on the first day are not available, in which 70.9% (180/254) in the early albumin combination group and 57.1% (761/1,332) in the crystalloid-only group. To reduce selective bias, we compared the relationships between early albumin administration and 30-day mortality in patients with no albumin deficiency on the day1, the results showed that there was no statistical significance on minimum albumin levels on 1st day (2.8 vs. 2.5 g/dl, *P* = 0.072) between survivors and non-survivors in early albumin combination group (Supplementary Table 3). However, in CS patients aged ≥60 years with no albumin deficiency on day1, compared with the crystalloid-only group, we found early albumin combination was associated with reduced 30-day mortality (54 vs. 33%, *P* = 0.035) and 60-day mortality (57 vs. 35%, *P* = 0.036; Supplementary Table 4).

**Figure 2:**
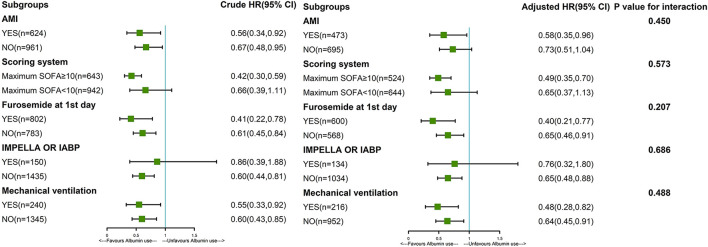
Subgroup analysis of the association between 30-day mortality and early albumin use. The association between early combination treatment and mortality remained significant in other subgroups such as maximum SOFA ≥10, without IMPELLA or IABP, and with or without furosemide and mechanical ventilation. Nonetheless, the association was not significant in IMPELLA or IABP and maximum SOFA <10 subgroups. Patients with AMI (HR = 0.58) showed a 42% reduction in 30-day mortality risk in the early albumin group than the crystal-only group. Patients without AMI (HR = 0.73) showed that the risk of death at 30 days was 27% lower in the early albumin group than in the crystal-only group. In the last column, P for interaction was 0.450, indicating that there was no significant difference in the relationship between albumin treatment and death between patients with and without AMI. That is to say, the previous differences of 0.58 and 0.73 in HR were not significant, and the relationship between albumin treatment and death at 30 days could not be considered to be different in patients with and without AMI. Other parameters like scoring system, IMPELLA or IABP, furosemide, and mechanical ventilation are interpreted similarly to AMI.

**Figure 3:**
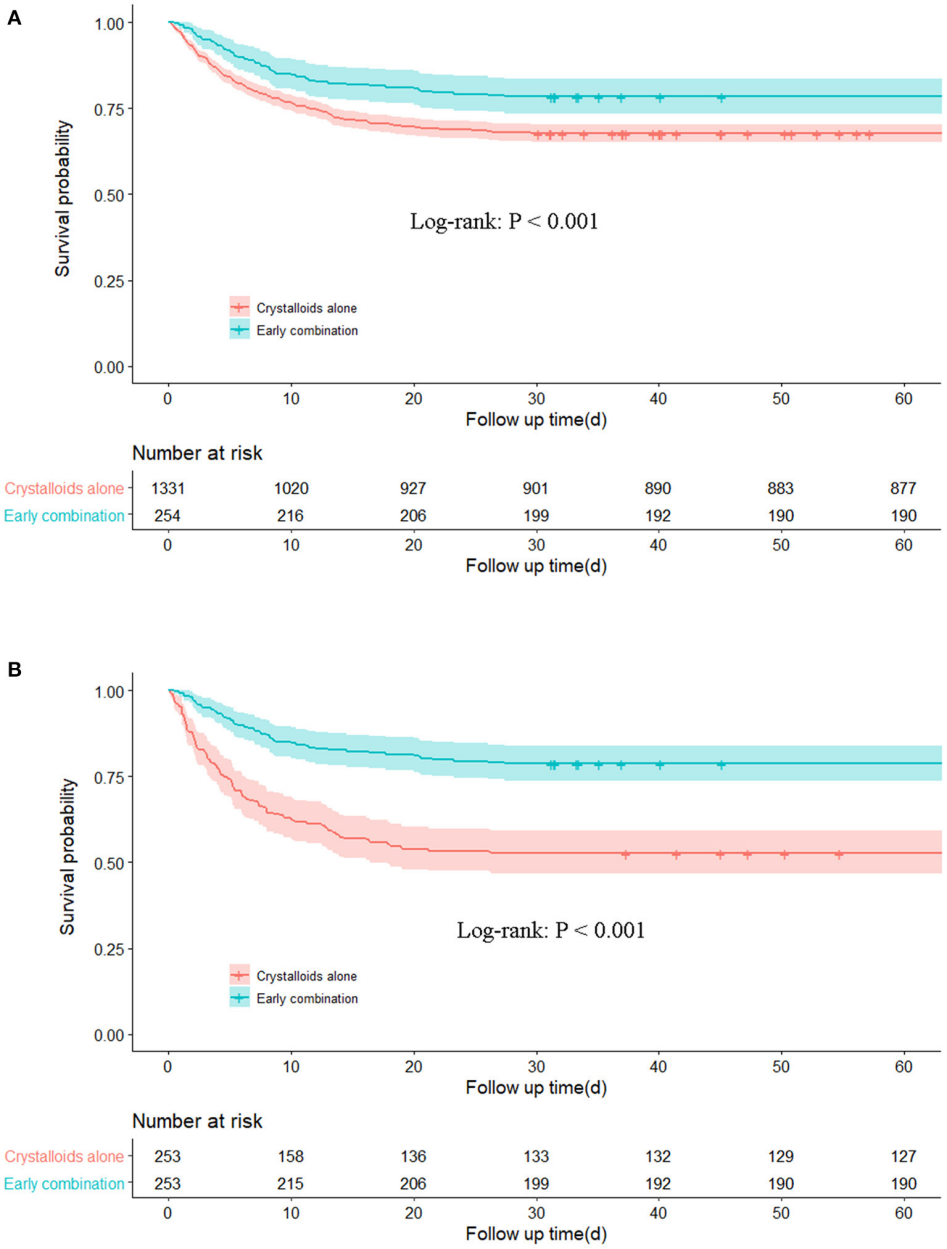
Kaplan–Meier survival curve of two groups before **(A)** and after **(B)** PSM.

Subgroup analysis was performed for AMI, maximum SOFA, IMPELLA or IABP, furosemide, and mechanical ventilation (Figure 2). Early combination treatment was significantly associated with reduced 30-day mortality in the AMI subgroup (adjusted HR 0.58, 95% CI 0.35–0.96) but not in the non-AMI subgroup. The association between early combination treatment and 30-day mortality remained significant in other subgroups such as maximum SOFA ≥10, without IMPELLA or IABP, and with or without furosemide and mechanical ventilation. Nonetheless, the association was not significant in IMPELLA or IABP and maximum SOFA <10 subgroups. Moreover, K–M curves showed a significant difference between the two groups before and after PSM (*P* < 0.001; Figures 3A,B). Smooth curve fitting showed that protein levels at 24 and 48 h after admission correlated with 30-day mortality, with higher mortality with lower albumin levels (Supplementary Figures 2, 3).

## Discussion

This study demonstrates that in CS, resuscitation with combination albumin within 24 h is associated with decreased 30-day and 60-day mortality (21.7 vs. 32.4% and 25.2 vs. 34.2%, respectively), prolonging the length of stay in the ICU (10.61 vs. 5.96 days) and increasing the incidence of mechanical ventilation (90.1 vs. 65.6%,). Additionally, this result is stable in PSM analysis after adjusting for covariates. Results for subgroups of maximum SOFA ≥10, with AMI, without IMPELLA or IABP, and with or without furosemide and mechanical ventilation were robust. However, the association is not significant in maximum SOFA <10, IMPELLA or IABP, and non-AMI subgroups. Our results suggest that early albumin combination therapy has a viable beneficial effect in patients aged ≥60 years with CS, which has not been previously reported.

There is still great controversy about the use of albumin for resuscitation in shock. Hill et al. (19) and Martin et al. (20) found that albumin administration increased the risk of pulmonary edema. Compared with crystalloids, most studies have shown that albumin has no effect on the outcome in critically ill patients (5, 21) and has no effect on pulmonary edema (22), and many studies have been carried out to further investigate the therapeutic effect of albumin. Indeed, comparisons of albumin and crystals have been studied in critically ill patients (23), sepsis (7), septic shock (24), non-cardiac surgery (21), hemorrhagic shock (25), and stroke (20). Nevertheless, some studies have shown that albumin can help to improve outcomes (24) and reduce multiple organ failure (26). In critically ill patients who need large-volume resuscitation (>60 ml/kg within 24 h), 5% albumin was associated with reduced mortality and major adverse kidney events at 30, 90, and 365 days (27). Therefore, due to the heterogeneity of shock patients, albumin use in appropriate patients may improve outcomes.

Based on its pathophysiological characteristics, clinical shock can be divided into distributed shock, hypovolemic shock, obstructive shock, and cardiogenic shock. The latest sepsis guidelines recommend the use of albumin for patients undergoing massive crystalloid resuscitation, but there is no recommendation for CS. The causes of CS may be insufficient effective circulation due to coronary artery stenosis, myocarditis, dystonia or dysfunction of the conduction bundle and peripheral blood vessels. Proper fluid resuscitation is an important approach for correcting insufficient circulation, but due to the high osmotic pressure of albumin, there may be subsequent fluid overload and pulmonary edema. Thus, rapid albumin infusion has become a relative contraindication of CS in primary health care without sufficient ability to monitor pulmonary oedema. Most guidelines, including in Europe (4, 15), the United States (1, 2), and France (28), indicate that in the absence of pulmonary edema, hypovolemic CS patients require resuscitation therapy, but most guidelines do not make specific recommendations with regard to the type of fluid. The 2016 ESC guidelines (15) recommend the use of normal saline or sodium lactate Ringer's solution(>200 ml/15–30 min), but do not list evidence. Tewelde et al. (1) pointed out that crystal fluid for rescue treatment is recommended, similar to septic shock. However, there are significant differences in etiology and pathology between the two types of shock, and it is necessary to determine whether albumin can be used for fluid resuscitation of CS.

The main indication of albumin is hypoproteinaemia (albumin level below 3.0 g/dl). Hypoproteinaemia has been shown to be closely related not only to the mortality of patients with trauma (29) and other severe cases (30) but also to acute myocardial infarction (31) and acute or chronic heart failure (32). Hypoproteinaemia is also an independent risk factor for poor prognosis in CS (33, 34). In our study, we analyzed the effect of albumin infusion on the prognosis of patients with different albumin levels in two groups and found that levels at 24 and 48 h after admission correlated with 30-day mortality and that the level was lower and mortality higher, especially at 24 h. In the study, the SOFA score on the first day was high, the albumin level low, the central venous pressure (CVP) high, and the 30-day mortality low in the combined albumin group. After combined albumin resuscitation, the CVP was still high, but there was no significant difference in plasma albumin level between the two groups; even after PSM, the minimum albumin level on the second day was higher in the early combination group than in the crystalloid alone group. Moreover, 30-day mortality was significantly lower than that in the crystal fluid resuscitation-only group. Therefore, we used the *E*-value to assess whether unmeasured protective factors influence existing outcomes, indicating that early combined albumin resuscitation can reduce the 30-day mortality of CS patients.

This study has several limitations. First, we failed to extract hypovolemic status and pulmonary oedema upon ICU admission. Although we sought to adjust confounding factors and minimize potential bias, the disadvantages of a retrospective study cannot be avoided. A rigorously designed randomized controlled study may be the only way to resolve the imbalance between the groups. Second, we deduced that the use of albumin might decrease mortality in this specific cohort. However, the adverse effects of albumin, such as aggravating pulmonary edema, should also be considered. Overall, the findings should be interpreted with caution, especially for patients with no shortage of volume or volume overload. Third, the missing albumin level and CVP value data were larger than 40% and we did not perform multiple imputations. However, the association between early albumin combination and 30-day mortality was further confirmed by PSM analysis in CS patients aged≥60 with no albumin deficiency on day 1. Finally, although PSM may minimize possible confounders, it also significantly reduced the sample size of our study population, compared with the original data, the distribution of the matched dataset was smaller. Therefore, the results should be interpreted with caution. Further prospective research is needed to verify our findings.

## Conclusions

Early resuscitation with combination albumin is associated with lower 30-day mortality in patients with CS aged ≥60, especially in patients with maximum SOFA≥10, AMI, without Impella or IABP, and with or without furosemide and mechanical ventilation. In the future, more extensive randomized clinical trials are needed to confirm and verify this association.

## Data Availability Statement

The original contributions presented in the study are included in the article/Supplementary Material, further inquiries can be directed to the corresponding authors.

## Ethics Statement

The studies involving human participants were reviewed and approved by the Research Ethics Committee of Shenzhen Second People's Hospital. Written informed consent for participation was not required for this study in accordance with the national legislation and the institutional requirements.

## Author Contributions

MW and Z-yZ were responsible for the study concept and design. Z-yZ and BW were responsible for collecting the data. Z-yZ was responsible for the statistical analysis. Y-yL and Z-yZ were responsible for drafting the manuscript. W-jP, Z-pZ, J-jH, Z-jY, and J-jZ were responsible for literature retrieval. MW and BC was responsible for critical reading of a final version of the manuscript. All authors had full access to all the data in the study, take responsibility for the integrity of the data, and the accuracy of the data analysis.

## Funding

This work was supported by grants from the Sanming Project of Medicine in Shenzhen (SZSM20162011), Shenzhen Science and Technology Innovation Commission (JCYJ20190806163603504), and Shenzhen Second People's Hospital Clinical Research Fund of Guangdong Province High-level Hospital Construction Project (20173357201815, 20193357003, and 20203357014).

## Conflict of Interest

The authors declare that the research was conducted in the absence of any commercial or financial relationships that could be construed as a potential conflict of interest.

## Publisher's Note

All claims expressed in this article are solely those of the authors and do not necessarily represent those of their affiliated organizations, or those of the publisher, the editors and the reviewers. Any product that may be evaluated in this article, or claim that may be made by its manufacturer, is not guaranteed or endorsed by the publisher.

## References

[1] Tewelde SZ Liu SS Winters ME. Cardiogenic shock. Cardiol Clin. (2018) 36:53–61. 10.1016/j.ccl.2017.08.009 29173681

[2] van Diepen S Katz JN Albert NM Henry TD Jacobs AK Kapur NK. Contemporary management of cardiogenic shock: a scientific statement from the American Heart Association. Circulation. (2017) 136:e232–68. 10.1161/CIR.0000000000000525 28923988

[3] Hochman JS Sleeper LA Webb JG Sanborn TA White HD Talley JD. Early revascularization in acute myocardial infarction complicated by cardiogenic shock. SHOCK investigators should we emergently revascularize occluded coronaries for cardiogenic shock. N Engl J Med. (1999) 341:625–34. 10.1056/NEJM199908263410901 10460813

[4] Thiele H Ohman EM de Waha-Thiele S Zeymer U Desch S. Management of cardiogenic shock complicating myocardial infarction: an update 2019. Eur Heart J. (2019) 40:2671–83. 10.1093/eurheartj/ehz363 31274157

[5] Caironi P Tognoni G Masson S Fumagalli R Pesenti A Romero M. Albumin replacement in patients with severe sepsis or septic shock. N Engl J Med. (2014) 370:1412–21. 10.1056/NEJMoa1305727 24635772

[6] Finfer S Bellomo R Boyce N French J Myburgh J Norton R. comparison of albumin and saline for fluid resuscitation in the intensive care unit. N Engl J Med. (2004) 350:2247–56. 10.1056/NEJMoa040232 15163774

[7] Zhou S Zeng Z Wei H Sha T An S. Early combination of albumin with crystalloids administration might be beneficial for the survival of septic patients: a retrospective analysis from MIMIC-IV database. Ann Intensive Care. (2021) 11:42. 10.1186/s13613-021-00830-8 33689042 PMC7947075

[8] Evans L Rhodes A Alhazzani W Antonelli M Coopersmith CM French C. Surviving sepsis campaign: international guidelines for management of sepsis and septic shock 2021. Intensive Care Med. (2021) 47:1181–247. 10.1007/s00134-021-06506-y 34599691 PMC8486643

[9] Fanali G di Masi A Trezza V Marino M Fasano M Ascenzi P. Human serum albumin: from bench to bedside. Mol Aspects Med. (2012) 33:209–90. 10.1016/j.mam.2011.12.002 22230555

[10] Evans TW. Review article: albumin as a drug–biological effects of albumin unrelated to oncotic pressure. Aliment Pharmacol Ther. (2002) 16 Suppl 5:6–11. 10.1046/j.1365-2036.16.s5.2.x 12423448

[11] Belinskaia DA Voronina PA Shmurak VI Jenkins RO Goncharov NV. Serum albumin in health and disease: esterase, antioxidant, transporting and signaling properties. Int J Mol Sci. (2021) 22:10318. 10.3390/ijms221910318 34638659 PMC8508759

[12] Johnson A Bulgarelli L Pollard T Horng S Celi LA Mark R. MIMIC-IV (version 1.0). PhysioNet [Internet] (2021). 10.13026/s6n6-xd98

[13] Khwaja A. KDIGO Clinical practice guidelines for acute kidney injury. Nephron. (2012) 120:c179–c84. 10.1159/000339789 22890468

[14] Thygesen K Alpert JS Jaffe AS Chaitman BR Bax JJ Morrow DA. Fourth universal definition of myocardial infarction. J Am Coll Cardiol. (2018) 72:2231–64. 10.1016/j.jacc.2018.08.1038 30153967

[15] Ponikowski P Voors AA Anker SD Bueno H Cleland JG Coats AJ. 2016 ESC guidelines for the diagnosis and treatment of acute and chronic heart failure: the Task Force for the diagnosis and treatment of acute and chronic heart failure of the European Society of Cardiology (ESC) Developed with the special contribution of the Heart Failure Association (HFA) of the ESC. Eur J Heart Fail. (2016) 18:891–975. 10.1002/ejhf.592 27207191

[16] Haneuse S VanderWeele TJ Arterburn D. Using the E-value to assess the potential effect of unmeasured confounding in observational studies. JAMA. (2019) 321:602–3. 10.1001/jama.2018.21554 30676631

[17] Zhang Z Kim HJ Lonjon G Zhu Y. Balance diagnostics after propensity score matching. Ann Transl Med. (2019) 7:16. 10.21037/atm.2018.12.10 30788363 PMC6351359

[18] Pedersen AB Mikkelsen EM Cronin-Fenton D Kristensen NR Pham TM Pedersen L. Missing data and multiple imputation in clinical epidemiological research. Clin Epidemiol. (2017) 9:157–66. 10.2147/CLEP.S129785 28352203 PMC5358992

[19] Hill MD Martin RH Palesch YY Moy CS Tamariz D Ryckborst KJ. Albumin administration in acute ischemic stroke: safety analysis of the ALIAS part 2 multicenter trial. PLoS ONE. (2015) 10:e0131390. 10.1371/journal.pone.0131390 26325387 PMC4556660

[20] Martin RH Yeatts SD Hill MD Moy CS Ginsberg MD Palesch YY. (Albumin in Acute Ischemic Stroke) trials: analysis of the combined data from parts 1 and 2. Stroke. (2016) 47:2355–9. 10.1161/STROKEAHA.116.012825 27462118 PMC4995121

[21] Tyagi A Maitra S Bhattacharjee S. Comparison of colloid and crystalloid using goal-directed fluid therapy protocol in non-cardiac surgery: a meta-analysis of randomized controlled trials. J Anesth. (2020) 34:865–75. 10.1007/s00540-020-02832-5 32719939

[22] van der Heijden M Verheij J van Nieuw Amerongen GP Groeneveld AB. Crystalloid or colloid fluid loading and pulmonary permeability, edema, and injury in septic and nonseptic critically ill patients with hypovolemia. Crit Care Med. (2009) 37:1275–81. 10.1097/CCM.0b013e31819cedfd 19242338

[23] Lewis SR Pritchard MW Evans DJ Butler AR Alderson P Smith AF. Colloids versus crystalloids for fluid resuscitation in critically ill people. Cochrane Database Syst Rev. (2018) 8:Cd000567. 10.1002/14651858.CD000567.pub7 30073665 PMC6513027

[24] Hariri G Joffre J Deryckere S Bigé N Dumas G Baudel JL. Albumin infusion improves endothelial function in septic shock patients: a pilot study. Intensive Care Med. (2018) 44:669–71. 10.1007/s00134-018-5075-2 29392345

[25] Torres LN Salgado CL Dubick MA Cap AP Torres Filho IP. Role of albumin on endothelial basement membrane and hemostasis in a rat model of hemorrhagic shock. J Trauma Acute Care Surg. (2021) 91(2S Suppl 2):S65–73. 10.1097/TA.0000000000003298 34039924

[26] Joosten A Delaporte A Ickx B Touihri K Stany I Barvais L. Crystalloid versus colloid for intraoperative goal-directed fluid therapy using a closed-loop system: a randomized, double-blinded, controlled trial in major abdominal surgery. Anesthesiology. (2018) 128:55–66. 10.1097/ALN.0000000000001936 29068831

[27] Gomez H Priyanka P Bataineh A Keener CM Clermont G Kellum JA. Effects of 5% albumin plus saline versus saline alone on outcomes from large-volume resuscitation in critically ill patients. Crit Care Med. (2021) 49:79–90. 10.1097/CCM.0000000000004706 33165027 PMC7746571

[28] Levy B Bastien O Karim B Cariou A Chouihed T Combes A. Experts' recommendations for the management of adult patients with cardiogenic shock. Ann Intensive Care. (2015) 5:52. 10.1186/s13613-015-0052-1 26152849 PMC4495097

[29] Sung J Bochicchio GV Joshi M Bochicchio K Costas A Tracy K. Admission serum albumin is predicitve of outcome in critically ill trauma patients. Am Surg. (2004) 70:1099–102. 15663053

[30] McCluskey A Thomas AN Bowles BJ Kishen R. The prognostic value of serial measurements of serum albumin concentration in patients admitted to an intensive care unit. Anaesthesia. (1996) 51:724–7. 10.1111/j.1365-2044.1996.tb07883.x 8795312

[31] Oduncu V Erkol A Karabay CY Kurt M Akgün T Bulut M. The prognostic value of serum albumin levels on admission in patients with acute ST-segment elevation myocardial infarction undergoing a primary percutaneous coronary intervention. Coron Artery Dis. (2013) 24:88–94. 10.1097/MCA.0b013e32835c46fd 23249632

[32] Liu M Chan CP Yan BP Zhang Q Lam YY Li RJ. Albumin levels predict survival in patients with heart failure and preserved ejection fraction. Eur J Heart Fail. (2012) 14:39–44. 10.1093/eurjhf/hfr154 22158777

[33] Huang M Ong BH Hoo AEE Gao F Chao VTT Lim CH. Prognostic factors for survival after extracorporeal membrane oxygenation for cardiogenic shock. ASAIO J. (2020) 66:141–5. 10.1097/MAT.0000000000000984 30864968

[34] Jäntti T Tarvasmäki T Harjola VP Parissis J Pulkki K Javanainen T. Hypoalbuminemia is a frequent marker of increased mortality in cardiogenic shock. PLoS ONE. (2019) 14:e0217006. 10.1371/journal.pone.0217006 31095609 PMC6522037

