# Supplementary material for: Early Combination of Albumin With Crystalloid Administration Might Reduce Mortality in Patients With Cardiogenic Shock: An Over 10-Year Intensive Care Survey
Source: Front Cardiovasc Med. 2022 May 27;9:879812. doi: 10.3389/fcvm.2022.879812 (PMC9184452; doi:10.3389/fcvm.2022.879812)
Supplement: Supplementary file 1 [file Data_Sheet_1.pdf]

Supplementary materials: tables and figures

Table S1. Missing number (%) for risk variables and outcome variables

Risk variables	Missing number (%)
Age	0(0)
Male	0(0)
White	0(0)
Insurance, Medicare	0(0)
Weighta	18(1.13%)
AMI	0(0)
AHF	0(0)
Hypertension	0(0)
Diabetes	0(0)
Chronic pulmonary disease	0(0)
CKD	0(0)
Stroke	0(0)
Minimum SBPa	15(0.95%)
Minimum DBPa	15(0.95%)
CCI	0(0)
Maximum SOFA at 1st day	0(0)
Maximum SAPS II at 1st day	0(0)
Minimum Albumin at 1st dayb	941(59.33%)
Minimum Albumin at 2nd dayb	1288(81.21%)
Maximum CVP at 1st dayb	765(48.23%)
Maximum CVP at 2nd dayb	752(47.41%)
PCI	0(0)
IABP	0(0)
IMPELLA	0(0)
Furosemide at 1st day	0(0)
Dopamine at 1st day	0(0)
Duration of dopamineb	1270(80.08%)
Norepinephrine at 1st day	0(0)
Duration of norepinephrineb	745(46.97%)
Outcome variables	
Urine output (ml/1st 24hr), median(IQR)b	60(3.78%)
Volume of crystalloids (ml/1st 24hr), median (IQR)	0(0)
ICU LOS (days), mean (SD)	0(0)
Hospital LOS (days), mean (SD)	0(0)
AKI, n (%)	0(0)
AKI stage 3, n (%)	0(0)
RRT at 1st day, n (%)	0(0)
Mechanical ventilation, n (%)	0(0)
30-day mortality, n (%)	0(0)
60-day mortality, n (%)	0(0)

Abbreviations: AMI: acute myocardial infarction; AHF: acute heart failure; CKD: chronic kidney disease; SBP: systolic blood pressure; DBP: diastolic blood pressure; CCI: charlson comorbidity index; SOFA: sequential organ failure assessment; SAPS II: Simplified acute physiology score II; CVP: central venous pressure; PCI: percutaneous coronary intervention; IABP: intra-aortic balloon pump; ICU: intensive care

unit; LOS: length of stay; AKI: acute kidney injury; RRT: renal replacement therapy.
[a]The proportion of missing values is less than 2%. Averages were used to replace these missing values;
[b]The proportion of missing values is larger than 40%. Those variables were not included in multivariate analysis and PSM analysis.

Table S2. Risk factors of 30-day mortality in CS patients after PSM

Variables	HR	95% CI	P value
Early combination	0.390	0.282-0.539	<0.001
Age	1.009	0.997-1.02	0.129
Male	0.936	0.684-1.279	0.677
White	0.963	0.6981-1.327	0.816
Insurance, Medicare	1.359	0.9984-1.849	0.051
Weight	0.996	0.989-1.003	0.291
AMI	1.371	1.009-1.863	0.044
AHF	0.625	0.449-0.870	0.005
Hypertension	0.916	0.614-1.367	0.666
Diabetes	1.356	0.999-1.840	0.0508
Chronic pulmonary disease	1.153	0.839-.583	0.384
CKD	1.236	0.908-1.683	0.178
Stroke	1.363	0.931-1.994	0.123
Minimum SBP	0.973	0.963-0.982	<0.001
Minimum DBP	0.975	0.961-0.989	<0.001
CCI	1.099	1.033-1.168	0.003
Maximum SOFA at 1st day	1.146	1.099-1.196	<0.001
Maximum SAPS II at 1st day	1.043	1.034-1.053	<0.001
Minimum Albumin at 1st day	0.673	0.478-0.949	0.0237
Minimum Albumin at 2nd day	0.656	0.376-1.146	0.138
Maximum CVP at 1st day	0.999	0.967-1.031	0.927
Maximum CVP at 2nd day	1.022	0.991-1.054	0.172
PCI	0.903	0.602-1.356	0.624
IABP	2.162	1.173-3.987	0.0135
IMPELLA	1.88	1.067- 3.313	0.029
Furosemide at 1st day	0.7135	0.502-1.015	0.0605
Dopamine at 1st day	1.726	0.959-3.106	0.0687
Norepinephrine at 1st day	1.84	1.245-2.718	0.0022

Abbreviation: IQR interquartile range; AMI: acute myocardial infarction; AHF: acute heart failure; CKD: chronic kidney disease; SBP: systolic blood pressure; DBP: diastolic blood pressure; CCI: charlson comorbidity index; SOFA: sequential organ failure assessment; SAPS II: Simplified acute physiology score II; CVP: central venous pressure; PCI: percutaneous coronary intervention; IABP: intra-aortic balloon pump.

Table S3 Comparisons of baseline characteristics and outcomes between survivors and non-survivors in early albumin combination groups

Variables	Total(n = 254)	30-day survivors（n = 199）	30-day non-survivors（n = 55）	P-value
**Baseline characteristics**				
Age(year), median (IQR)	71.4 (61.8, 78.2)	72.3 (62.4, 78.3)	70.8 (59.2, 77.9)	0.30
male, n (%)	161 (63.4)	129 (64.8)	32 (58.2)	0.37
White, n (%)	172 (67.7)	135 (67.8)	37 (67.3)	0.94
Insurance, Medicare, n (%)	136 (53.5)	102 (51.3)	34 (61.8)	0.16
Weight, median(IQR)	80.0 (69.5, 91.1)	80.0 (70.0, 91.1)	75.0 (65.9, 94.5)	0.18
Etiology, n (%)				
AMI	92 (36.2)	69 (34.7)	23 (41.8)	0.33
AHF	98 (38.6)	82 (41.2)	16 (29.1)	0.10
History of disease, n (%)				
Hypertension	216 (85.0)	169 (84.9)	47 (85.5)	0.92
Diabetes	98 (38.6)	76 (38.2)	22 (40.0)	0.81
Chronic pulmonary disease	78 (30.7)	58 (29.1)	20 (36.4)	0.30
CKD	89 (35.0)	70 (35.2)	19 (34.5)	0.93
Stroke	41 (16.1)	32 (16.1)	9 (16.4)	0.96
Vital signs at 1st day, median(IQR)				
Minimum SBP (mmHg)	80.0 (70.0, 85.5)	81.0 (73.0, 87.0)	72.0 (62.0, 81.0)	<0.001
Minimum DBP (mmHg)	42.0 (36.0, 48.0)	42.0 (37.0, 48.0)	39.0 (34.0, 46.0)	0.035
Scoring system, mean (SD)				
CCI	6.5 (2.4)	6.5 (2.4)	6.6 (2.5)	0.61
Maximum SOFA at 1st day	10.4 (3.5)	10.1 (3.4)	11.4 (3.6)	0.014
Maximum SAPS II at 1st day	47.5 (12.7)	46.1 (12.3)	52.8 (13.0)	<0.001
Laboratory findings				
Minimum Albumin at 1st day(g/dl), mean (SD)	2.7 (0.8)	2.8 (0.7)	2.5 (0.8)	0.072
Maximum CVP at 1st day(mmHg), median (IQR)	23.0 (20.0, 27.0)	22.0 (20.0, 26.0)	24.0 (20.0, 30.0)	0.37
In-hospital management, n (%)				
PCI	46 (18.1)	32 (16.1)	14 (25.5)	0.11
IABP	9 (3.5)	6 (3.0)	3 (5.5)	0.39
IMPELLA	13 (5.1)	7 (3.5)	6 (10.9)	0.028
In-hospital medication				
Furosemide at 1st day, n (%)	79 (31.1)	69 (34.7)	10 (18.2)	0.019
Dopamine at 1st day, n (%)	13 (5.1)	6 (3.0)	7 (12.7)	0.004
Duration of dopamine(h), median (IQR)	13.0 (3.5, 49.0)	10.0 (3.0, 59.0)	18.0 (5.0, 39.0)	0.73
Norepinephrine at 1st day, n (%)	183 (72.0)	139 (69.8)	44 (80.0)	0.14
Duration of norepinephrine(h), median (IQR)	46.0 (22.0, 95.0)	44.0 (19.0, 70.0)	59.0 (26.0, 140.0)	0.043
**Clinical outcomes**				
Minimum Albumin at 2nd day(g/dl), mean (SD)	3.0 (0.6)	3.1 (0.6)	2.9 (0.6)	0.26

Maximum CVP at 2nd day(mmHg), median (IQR)	24.0 (20.0, 29.0)	22.5 (20.0, 26.0)	26.0 (24.0, 30.0)	<0.001
Urine output (ml/1st 24hr), median(IQR)	1280.0 (704.5, 2025.0)	1360.0 (868.0, 2060.0)	845.0 (499.0, 1675.0)	0.007
Volume of crystalloid (ml/1st 24hr), median (IQR)	4409.2 (2621.7, 6682.5)	4232.2 (2603.9, 6377.2)	4812.6 (2773.0, 9152.1)	0.12
ICU LOS (days), mean (SD)	10.61 (11.34)	11.67 (12.31)	6.79 (5.29)	0.005
Hospital LOS (days), mean (SD)	17.7 (17.4)	20.1 (18.6)	8.9 (6.9)	<0.001
AKI, n (%)	196 (77.2)	149 (74.9)	47 (85.5)	0.098
AKI stage 3, n (%)	51 (20.1)	35 (17.6)	16 (29.1)	0.059
RRT at 1st day, n (%)	12 (4.7)	4 (2.0)	8 (14.5)	<0.001
Mechanical ventilation, n (%)	243 (95.7)	193 (97.0)	50 (90.9)	0.050

Abbreviation: IQR interquartile range; AMI: acute myocardial infarction; AHF: acute heart failure; CKD: chronic kidney disease; SBP: systolic blood pressure; DBP: diastolic blood pressure; CCI: charlson comorbidity index; SOFA: sequential organ failure assessment; SAPS II: Simplified acute physiology score II; CVP: central venous pressure; PCI: percutaneous coronary intervention; IABP: intra-aortic balloon pump; ICU: intensive care unit; LOS: length of stay; AKI: acute kidney injury; RRT: renal replacement therapy.

Table S4 Characteristics and outcomes of the CS patients aged ⩾60 with no albumin deficiency on day1 in two groups

		Propensity score matching					
		Before			After[$]		
Variables	Total population (n = 491)	Crystalloids only (n = 439)	Early combination (n = 52)	P value	Crystalloids only (n = 46)	Early combination (n = 46)	P value
Age(yr), median (IQR)	75.8 (67.8,	76.4 (68.1,	71.8 (66.4,	0.002	68.1 (64.0, 77.8)	71.8 (67.4,	0.30
male, n (%)	271 (55.2)	241 (54.9)	30 (57.7)	0.70	29 (63)	27 (59)	0.67
White, n (%)	327 (66.6)	290 (66.1)	37 (71.2)	0.46	31 (67)	32 (70)	0.82
Insurance, Medicare, n (%)	306 (62.3)	271 (61.7)	35 (67.3)	0.43	32 (70)	30 (65)	0.66
Weight(kg), median(IQR)	78.3 (65.0,	78.3 (65.0,	77.1 (66.5,	0.94	78.2 (62.6,	77.1 (65.9,	0.98
Etiology, n (%)							
AMI	246 (50.1)	228 (51.9)	18 (34.6)	0.018	16 (35)	17 (37)	0.83
AHF	322 (65.6)	304 (69.2)	18 (34.6)	<0.00	19 (41)	18 (39)	0.83
History of disease, n (%)							
Hypertension	381 (77.6)	336 (76.5)	45 (86.5)	0.10	36 (78)	39 (85)	0.42
Diabetes	212 (43.2)	194 (44.2)	18 (34.6)	0.19	22 (48)	17 (37)	0.29
Chronic pulmonary disease	171 (34.8)	154 (35.1)	17 (32.7)	0.73	14 (30)	15 (33)	0.82
CKD	225 (45.8)	206 (46.9)	19 (36.5)	0.16	18 (39)	18 (39)	1.00
Stroke	52 (10.6)	45 (10.3)	7 (13.5)	0.48	6 (13)	7 (15)	0.76
Vital signs at 1st day,							
Minimum SBP (mmHg)	79.0 (69.0,	79.0 (70.0,	77.3 (65.5,	0.095	75.0 (66.0, 81.5)	78.5 (66.0,	0.27
Minimum DBP (mmHg)	42.0 (33.0,	42.0 (34.0,	38.5 (32.0,	0.15	40.3 (27.0, 46.0)	41.5 (33.0,	0.56
Scoring system, mean (SD)							
CCI	7.50 (2.03)	7.58 (2.04)	6.79 (1.87)	0.008	7.07 (2.55)	6.96 (1.84)	0.82
Maximum SOFA at 1st day	9.10 (4.17)	8.82 (4.12)	11.48 (3.84)	<0.00	11.59 (4.49)	11.22 (3.90)	0.67
Maximum SAPS II at 1st day	49.42 (14.73)	49.20 (14.93)	51.27 (12.85)	0.34	52.63 (17.03)	51.00 (12.80)	0.60
Laboratory findings							
Minimum Albumin at 1st day(g/dl), mean (SD)	3.14 (0.58)	3.18 (0.53)	2.79 (0.83)	<0.00 1	2.87 (0.52)	2.86 (0.81)	0.95
Maximum CVP at 1st day(mmHg), median (IQR)	21.0 (20.0, 30.0)	20.0 (18.0, 30.0)	26.0 (20.0, 30.0)	0.008	24.0 (20.0, 28.0)	25.0 (20.0, 30.0)	0.61
In-hospital management, n (%)							
PCI	127 (25.9)	117 (26.7)	10 (19.2)	0.25	7 (15)	8 (17)	0.78
IABP	35 (7.1)	34 (7.7)	1 (1.9)	0.12	1 (2%)	1 (2)	1.00
IMPELLA	26 (5.3)	23 (5.2)	3 (5.8)	0.87	5 (11)	3 (7)	0.46
In-hospital medication							

Furosemide at 1st day, n (%)	260 (53.0)	247 (56.3)	13 (25.0)	<0.00	15 (33)	12 (26)	0.49
Dopamine at 1st day, n (%)	114 (23.2)	110 (25.1)	4 (7.7)	0.005	3 (7)	4 (9)	0.69
Duration of dopamine(h),	22.5 (4.0, 51.0)	24.0 (4.0,	12.0 (4.0,	0.60	3.5 (1.0, 99.0)	11.5 (3.5, 44.5)	0.44
Norepinephrine at 1st day, n	290 (59.1)	250 (56.9)	40 (76.9)	0.006	38 (83)	34 (74)	0.31
Duration of norepinephrine(h), median (IQR)	43.5 (18.0, 85.0)	41.0 (16.0, 83.0)	56.0 (29.0, 125.0)	0.025	60.0 (25.0, 110.0)	52.0 (29.0, 93.0)	0.65
Clinical outcomes							
30-day mortality, n (%)	193 (39.3)	177 (40.3)	16 (30.8)	0.18	25 (54)	15 (33)	0.035
60-day mortality, n (%)	202 (41.1)	185 (42.1)	17 (32.7)	0.19	26 (57)	16 (35)	0.036

Abbreviation: IQR interquartile range; AMI: acute myocardial infarction; AHF: acute heart failure; CKD: chronic kidney disease; SBP: systolic blood pressure; DBP: diastolic blood pressure; CCI: charlson comorbidity index; SOFA: sequential organ failure assessment; SAPS II: Simplified acute physiology score II; CVP: central venous pressure; PCI: percutaneous coronary intervention; IABP: intra-aortic balloon pump.

$ Propensity score matching (PSM) was selected to balance variables, including age, sex, ethnic group, insurance condition, weight, probable aetiology, history of the disease, CCI, vital signs on the first day, the first day of maximum SOFA and SAPS scores, minimum albumin at first day, and in-hospital management and medication.

Supplement

Sensitivity analysis

Early combination of albumin with crystalloids administration is associated with 30-day mortality（21.7% *vs.* 32.4%, P <0.001, the result was robustness after PSM (21.3% *vs.*44.7%, P<0.001). The E-Value was >3.22 (upper limit 5.65), meaning that residual confounding could explain the observed association if there exists an unmeasured covariate having a relative risk association⩾3.22 with both 30-day mortality and early combination of albumin with crystalloids administration.

Significant known and measured risk factors for in-hospital mortality within the multivariable Cox-proportional hazard model included without IABP (HR, 2.162 [95% CI, 1.173-3.987]), IMPELLA (HR, 1.88 [95% CI, 1.067- 3.313]), Norepinephrine at 1st day (HR, 1.84 [95% CI 1.245-2.718])(Table S2). Therefore, it is unlikely that an unmeasured or unknown confounder would have a substantially greater effect on 30-day mortality (relative risk exceeding 3.22) than these known risk factors

Figure S1. Standardized mean difference (SMD) of variables before and after PSM.

Abbreviation: IQR interquartile range; AMI: acute myocardial infarction; AHF: acute heart failure; CKD: chronic kidney disease; SBP: systolic blood pressure; DBP: diastolic blood pressure; CCI: charlson comorbidity index; SOFA: sequential organ failure assessment; SAPS II: Simplified acute physiology score II; CVP: central venous pressure; PCI: percutaneous coronary intervention; IABP: intra-aortic balloon pump; SMD: standardized mean difference.

Fig.S2 Association between min albumin at the first day and 30-day mortality. The dotted lines on both sides represent 95% confidence interval.

Abbreviation: IQR interquartile range; AMI: acute myocardial infarction; AHF: acute heart failure; CKD: chronic kidney disease; SBP: systolic blood pressure; DBP: diastolic blood pressure; CCI: charlson comorbidity index; SOFA: sequential organ failure assessment; SAPS II: Simplified acute physiology score II; CVP: central venous pressure; PCI: percutaneous coronary intervention; IABP: intra-aortic balloon pump; SMD: standardized mean difference.

Fig.S3 Association between min albumin at second day and 30-day mortality. The dotted lines on both sides represent 95% confidence interval.

Abbreviation: IQR interquartile range; AMI: acute myocardial infarction; AHF: acute heart failure; CKD: chronic kidney disease; SBP: systolic blood pressure; DBP: diastolic blood pressure; CCI: charlson comorbidity index; SOFA: sequential organ failure assessment; SAPS II: Simplified acute physiology score II; CVP: central venous pressure; PCI: percutaneous coronary intervention; IABP: intra-aortic balloon pump.
