# Supplementary figures and images for: Early Combination of Albumin With Crystalloid Administration Might Reduce Mortality in Patients With Cardiogenic Shock: An Over 10-Year Intensive Care Survey
Source: Front Cardiovasc Med. 2022 May 27;9:879812. doi: 10.3389/fcvm.2022.879812 (PMC9184452; doi:10.3389/fcvm.2022.879812)

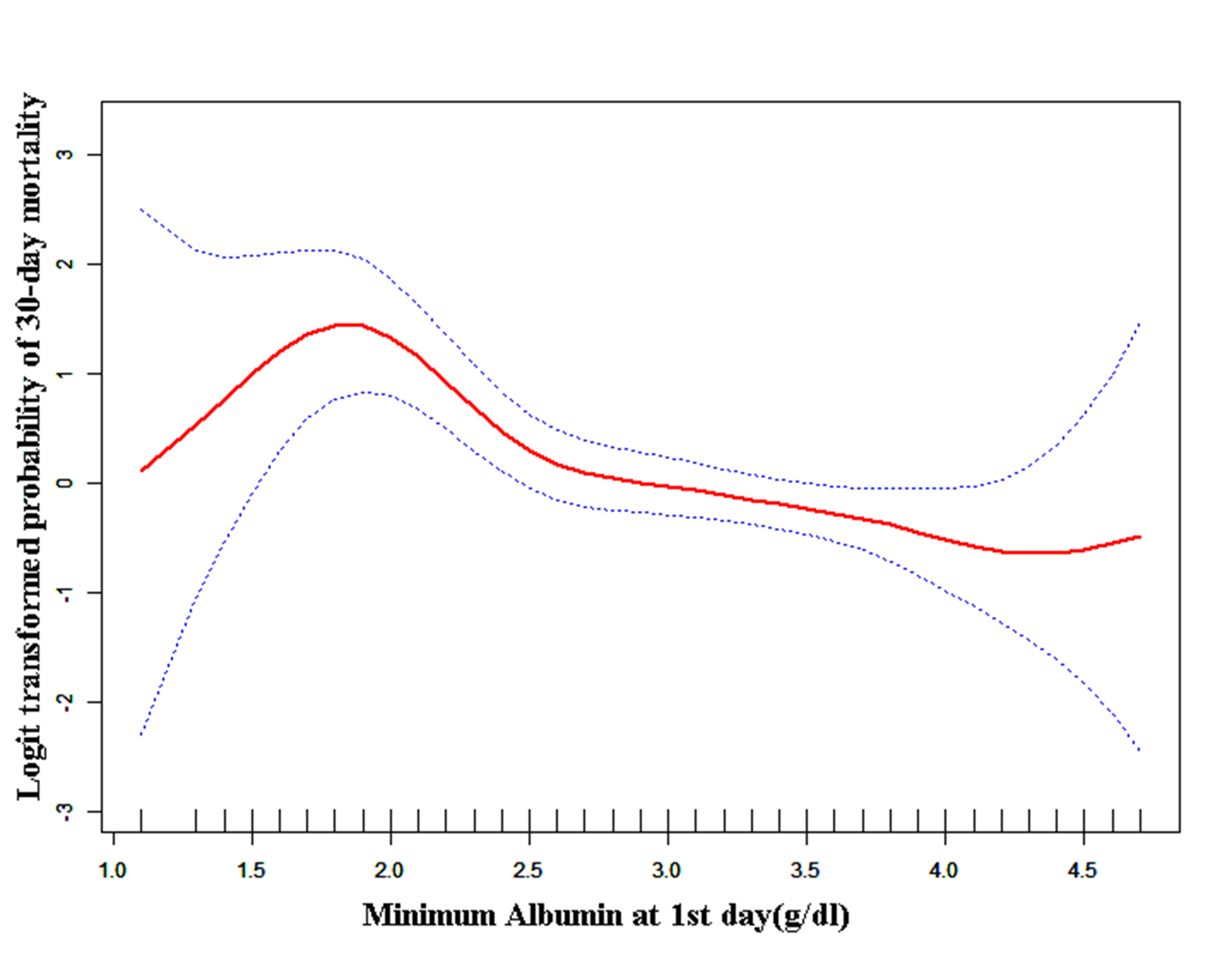

Supplement: Supplementary file 3 [file Image_2.tif]

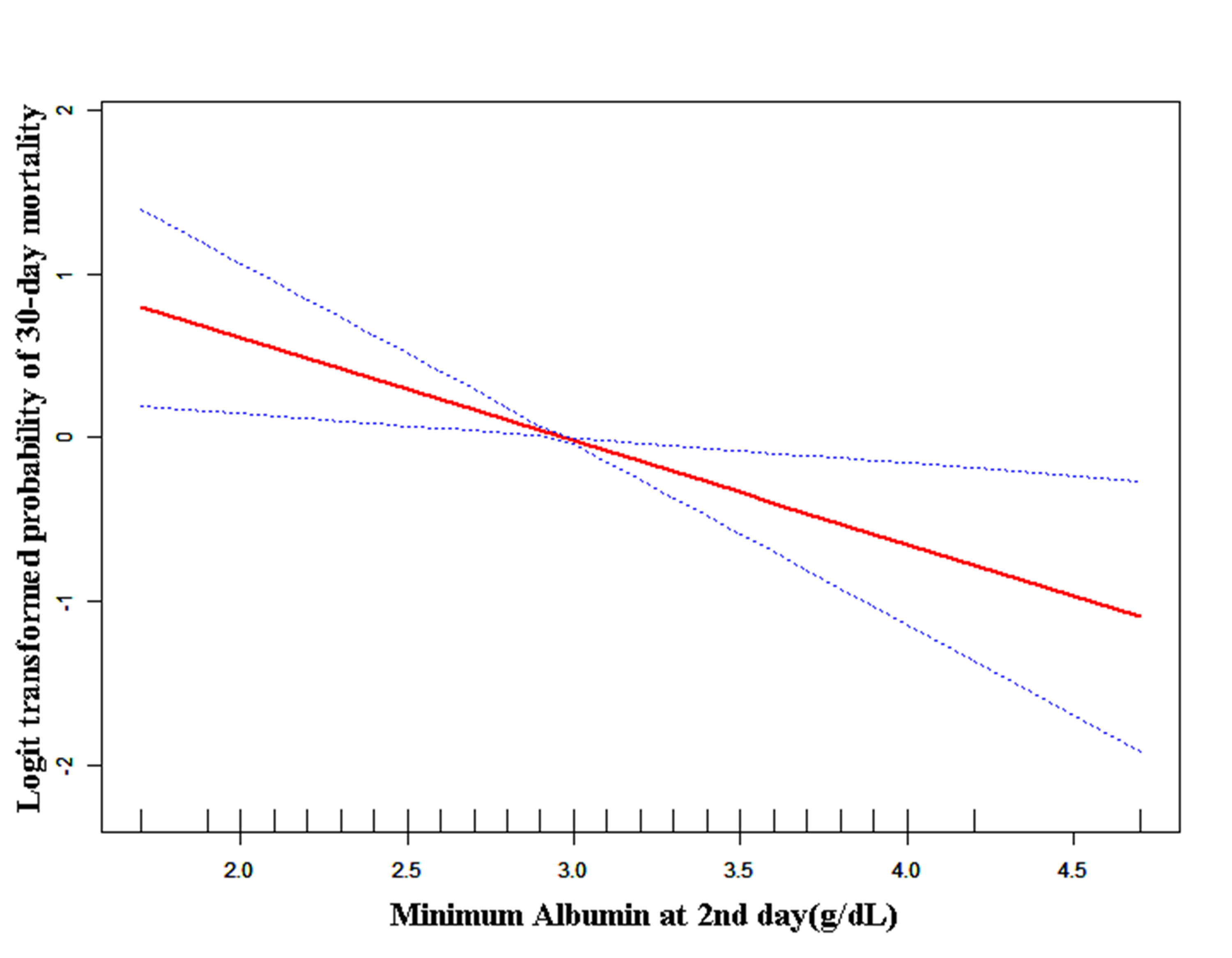

Supplement: Supplementary file 4 [file Image_3.tif]
